# Pharmacokinetics, safety and efficacy from randomized controlled trials of 1 and 2 mg nicotine bitartrate lozenges (Nicotinell^®^)

**DOI:** 10.1186/1472-6904-7-11

**Published:** 2007-10-08

**Authors:** Bertrand Dautzenberg, Mitchell Nides, Jean-Luc Kienzler, Anne Callens

**Affiliations:** 1 Groupe Hospitalier Pitié-Salpêtrière, Paris, France; 2 Los Angeles Clinical Trials, Los Angeles, CA, USA; 3 Novartis Consumer Health S.A., Nyon, Switzerland; 4 Novartis Santé Familiale, Rueil-Malmaison, France

## Abstract

**Background:**

The use of nicotine replacement therapy (NRT) can almost double the chances of success for smokers to quit. Nevertheless, there is still a considerable number of cessation attempts that are made without any treatment. This novel oral formulation, (lozenge containing nicotine bitartrate dihydrate) has been developed to enlarge the offer for efficient smoking cessation drug therapies, assuming that increasing treatment options will bring more smokers to find the support they personally need to stop smoking.

**Methods:**

Three pharmacokinetic (PK), one safety and two efficacy studies were carried out with Nicotinell lozenges. PK trials were: (1) a single-dose, three-way crossover study comparing 1 and 2 mg lozenges with 2 mg nicotine gum; (2) a multiple-dose, two-way crossover study comparing 1 mg lozenge with 2 mg gum; (3) a multiple-dose, three-way crossover study comparing 1 and 2 mg lozenges with 4 mg gum. Safety trial: (4) a single dose study to assess the safety of swallowing up to 12 lozenges containing 1 mg nicotine. Efficacy trials: two efficacy studies in (5) France and (6) the USA, including more than 900 smokers followed-up for up to one year, conducted with the 1 mg lozenge.

**Results:**

The results of the individual PK trials showed that the 1 mg Nicotinell lozenge is bioequivalent to 2 mg polacrilex gum, as demonstrated by similar blood PK parameters (t_max_, C_max_, AUC). The 2 mg lozenge was found to deliver quantities of nicotine that were intermediate between those delivered by 2 and 4 mg polacrilex gum.

The short-term efficacy of the 1 mg lozenge in comparison with placebo was also demonstrated with significantly more subjects continuously abstinent from smoking with active lozenges on week 6 in two different populations: moderate to heavy smokers (FTND between 4 and 7) OR = 1.72 [95% CI: 1.05–2.80]; heavy to very heavy smokers (FTND 6 and over) OR = 2.87 [95% CI: 1.18–6.97].

Nicotinell lozenges were found to be safe with mainly mild and reversible adverse events. The safety of the 1 mg lozenge formulation, even when misused was also demonstrated.

**Conclusion:**

The data presented in this review demonstrate high nicotine bioavailability, excellent safety profile and proven short-term efficacy of Nicotinell lozenges. At nominal equivalent doses 1 and 2 mg Nicotinell lozenges were shown to deliver larger amounts of bioavailable nicotine compared to the nicotine polacrilex gum. According to the data developed here, the systemic exposure to nicotine could be ranked: 4 mg polacrilex gum > 2 mg Nicotinell lozenge > 1 mg Nicotinell lozenge = 2 mg polacrilex gum.

Adverse events observed during the clinical trials were mild or moderate in severity, transient and completely reversible. With respect to efficacy in smoking cessation, significantly higher continuous abstinence rates were achieved with lozenge compared to placebo. In conclusion, Nicotinell lozenges offer a valuable addition to the therapeutic armamentarium available for smoking cessation.

## Background

Smoking is still the largest preventable cause of death and disease in the developed world and increases the risk for cancer, cardiovascular and lung diseases, among others [1-3]. Accordingly, smoking cessation provides immediate and lasting benefits to public health [4-6]. However, relatively few smokers succeed in quitting each year [7]. Nicotine replacement therapy (NRT) helps smokers quit by providing nicotine at levels usually lower than those obtained through smoking and without the toxins contained in tobacco smoke. NRT can reduce the craving for nicotine and the nicotine withdrawal symptoms which might otherwise jeopardize the smoking cessation efforts [8]. NRT has a well established success record [9] and its use is endorsed by health authorities [4,10-12].

Several forms of NRT are available for smokers to choose from, according to their specific needs and preferences [8,13]. Beside nicotine patches, which deliver nicotine slowly and continuously through the skin, a number of acute dosage forms have been developed, which allow smokers to self-administer the amount of nicotine needed, in order to achieve a consistent concentration of nicotine in the blood, and to ward off acute urges to smoke ('rescue medication' use) [14]. The first available acute dosage form was nicotine-polacrilex chewing-gum, which was launched in the 1980s. More recent additions are the vapor inhaler, sublingual tablet, lozenge (all for oral nicotine administration), and nasal spray (uptake via the nasal mucosa) [15].

The efficacy and safety of nicotine lozenges containing 2 or 4 mg of nicotine coupled to a polacrilex resin, from which it is released upon dissolution of the lozenge in the mouth, have been described and compared with nicotine-polacrilex gum also using a polacrilex resin [16]. The efficacy of the Nicotinell lozenges studied in this review has previously been described [17,18]. The versatility of lozenges has also been demonstrated in the treatment of smokers who had previously failed to quit on other NRT products, and of both very heavy and moderate/light smokers [19-21]. Indeed, when compared to the gum, lozenges are characterized by a number of advantages, such as fewer potential oral health limitations (e.g. dental works or temporomandibular joint pain), better social acceptance (e.g. at workplaces), and greater ease of use as they do not require chewing. As opposed to patches, lozenges provide all the advantages of acute dosage forms described above [22], including the possibility of self-adaptation of the dose and some degree of behavioral activity and sensory stimulation, which might be crucial for smokers trying to quit.

Despite considerable literature, there are few published studies directly comparing different NRT products [9]. In addition, pharmacokinetic (PK) data on nicotine lozenges in the literature remain scarce [23]. The present review provides the first comprehensive description of the PK profiles of 1 and 2 mg Nicotinell lozenges in comparison with 2 and 4 mg nicotine gum. In addition, the efficacy and safety of the 1 mg Nicotinell lozenge in smoking cessation are documented.

## Methods

### General

All presented studies were carried out in compliance with the Helsinki Declaration and its amendments and Good Clinical Practices. All protocols were accepted by local ethics committees (Table 1). All subjects provided written informed consent before enrolment in the studies.

**Table 1:** Ethic committee approvals

**Study**	**Ethical Committee**
- single-dose, comparing 1 and 2 mg lozenges with 2 mg gum - multiple-dose, comparing 1 mg lozenge with 2 mg gum	CCPPRB de Franche-Comté, France
- multiple-dose, comparing 1 and 2 mg lozenges with 4 mg gum	CCPPRB of the Hôtel-Dieu Hospital (Lyon – France)
- safety assessment of 1 mg lozenge after swallowing	CREC Regipharm (Brussels – Belgium)
- French efficacy trial	CCPPRB of the Pitié-Salpêtrière Hospital (Paris – France)
- US efficacy trial	IRB at Research Testing Laboratories, Inc. (Great Neck, NY, USA)

Participants to the studies were adult, healthy smokers usually smoking at least 20 cigarettes per day in the PK trials, or at least 10 cigarettes per day in the safety trial. During the PK and safety trials, participants were maintained under highly controlled conditions, including standardized food and fluid intake, restricted physical activity, and cigarette abstinence. Non-smoking compliance was controlled by measurement of carbon monoxide (CO) in expired air. Blood samples were collected at planned time-points. Concentrations of nicotine and cotinine in plasma or urine were determined by validated GC-MS methods with limits of quantification at respectively 1 ng/ml and 10 ng/ml for nicotine and cotinine (LC-MS/MS for the multiple-dose study comparing 1 and 2 mg lozenges with 4 mg gum). Residual nicotine content in the chewed gum (or sucked lozenge) after use was determined by HPLC.

### Single-dose pilot study comparing 1 and 2 mg lozenges with 2 mg gum

This was a single-center, open-label, single-dose, randomized, three-way, crossover pilot study in nine male volunteers to assess the PK parameters of 1 mg Nicotinell lozenges (containing 3.07 mg nicotine bitartrate dihydrate equivalent to 1 mg nicotine base), 2 mg Nicotinell lozenges (containing 6.134 mg nicotine bitartrate dihydrate equivalent to 2 mg nicotine base), and 2 mg nicotine gum (Nicorette^® ^gum; Pharmacia A.B.; containing 10 mg nicotine carboxylate cation resin equivalent to 2 mg nicotine base). The same brand of comparator gum was used throughout the ensuing clinical development. Subjects were randomized to one of the three groups and received one piece of trial medication during each of the three 32-hour observation periods, which always started with a 24-hour non-smoking phase, and were separated by 7-day washouts. Lozenges were sucked and gum chewed for 30 min according to a controlled schedule, and remainders were collected to measure drug delivery. In this pilot study, lozenges were not completely dissolved at the end of 30 min, therefore the sucking pattern was modified for subsequent studies. Blood was drawn before dosing and at 15, 30, 45 min, 1, 1.25, 1.5, 2, 3, 4, 6, and 8 h after dosing. Urine was collected during the 24 h run-in preceding administration and between 0–4 h and 4–8 h after dosing. Main criteria for comparison between different drugs were AUC_0–∞ _and C_max_, which were corrected for 100% drug delivery from lozenge. Ln-transformed values were submitted to 4-factor ANOVA (sequence, subject, period, treatment).

### Multiple-dose study comparing 1 mg lozenge with 2 mg gum

This was a single-center, open-label, multiple-dose, randomized, two-way, crossover PK study in 24 male volunteers that compared 1 mg Nicotinell lozenges with 2 mg nicotine polacrilex gum. Two 2-day observation periods were separated by a 7-day washout and each period started with a 22-hour run-in phase without smoking, followed by a 12-hour dosing phase and a 23-hour sampling phase. Twelve doses of nicotine were administered, i.e. one per hour from t = 0 to t = 11. Lozenges were sucked until complete dissolution, and the time needed to achieve this (usually about 30 min) was reported. Gum was chewed for 30 min according to a schedule monitored by a computer-controlled beeper, and residues were collected and frozen for HPLC analysis. Blood was drawn pre-dose, 15, 30 and 45 min after the first dose. In addition, blood was drawn before each new dose (at 1, 2, 3, 4, 5, 6, 7, 8, 9, 10, and 11 h) and after the last dose at 11.25, 11.5, 11.75, 12, 12.25, 12.5, 13, 13.5, 14, 15, 17, 19, 21, and 23 h (all times after first dose).

Main criteria for comparison of nicotine PK at steady state were C_max_, t_max _and AUC_11–12 _(AUC during one dosing interval). Secondary criteria were first dose data (C_max_, t_max_, AUC_0–1_), C_min _before each dose and t_1/2 _after the last dose. In addition, urinary and saliva pH variations were assessed and safety was controlled through assessment of cardiovascular parameters (blood pressure (BP), heart rate (HR), and ECG), standard laboratory tests and incidence of adverse events (AEs). C_max _and AUC_11–12_, ln-transformed values were submitted to 4-factor ANOVA (sequence, subject, period, treatment). A 90% confidence interval (CI) was constructed for the ratio (e.g. for lozenge/gum) of C_max _and AUC_11–12 _respectively. The primary criterion for relative bioequivalence was that the 90% confidence limits fall within the interval [0.80–1.25], i.e. the standard bioequivalence acceptance range (BAR). When appropriate, the enlarged BAR [0.7–1.43] was applied.

### Multiple-dose study comparing 1 and 2 mg lozenges with 4 mg gum

This was a single-center, open-label, multiple-dose, randomized, three-way, crossover PK study in 31 male volunteers. Its primary objectives were: (1) to confirm the dose-concentration proportionality between Nicotinell 1 and 2 mg lozenges and (2) to compare nicotine systemic exposure by Nicotinell 2 mg lozenge with that of Nicorette 4 mg gum. Secondary objectives were the completion of the PK profile of the 2 mg Nicotinell lozenge and the comparison of clinical safety of the three nicotine formulations. There were three treatment periods of 12 h (one for each product), separated by 7-day washout periods. Smoking was prohibited during the 24 h preceding treatment and until leaving the study center. During each treatment period, a total of twelve doses of nicotine were administered at a rate of 1 dose per hour. Lozenges were sucked until complete dissolution and gum were chewed for 30 min at 60 chews/min (timed using a calibrated metronome) while the subjects remained in a seated position. Blood samples for determination of plasma nicotine and cotinine concentrations were drawn pre-dose and at 7 and 9 h. After intake of the last dose (11 h), blood was drawn at 11.25, 11.5, 11.75, 12.5, 13, 14, 15, 17, 19, 21, and 23 h. Tolerability was assessed by spontaneous reporting of AEs.

Primary PK parameters for evaluation were steady state data after the last dose (AUC_11–12_, C_max _and t_max_). For the comparison between 1 and 2 mg lozenges (dose proportionality) and between 2 mg lozenge and 4 mg gum, ln-transformed values for C_max _and AUC_1112 _were submitted to 4-factor ANOVA (sequence, subject, period, treatment). Relative bioequivalence was determined by construction of 90% CIs and application of the standard BAR [0.80–1.25] and enlarged BAR [0.7–1.43] for the ratios of C_max _and AUC_11–12_, and by Friedman ANOVA for t_max_. In addition, complete safety and tolerability data were collected, including physical examination and vital signs, cardiovascular parameters, standard laboratory tests and incidence of AEs.

### Safety of 1 mg lozenge after swallowing

This was a single-center, open-label, escalating-dose, sequential group safety study with 24 volunteers of both sexes. The participants were divided into three groups of eight: subjects in group A swallowed three lozenges, in group B six lozenges, and in group C 12 lozenges. For every group, subjects were included in two steps: first, two volunteers were assessed, and if the administered dose was well tolerated the other six volunteers were included. Each dose level was followed by a 2-day observation period, including a 12 h period after dosing during which smoking was prohibited. Lozenges were swallowed as a single dose with 250 ml of water. Safety was assessed by examination of the plasma nicotine levels, subjects' clinical status, standard laboratory testing, gastric motility test (only the first two subjects of each group) and AEs reporting. Blood for determination of plasma nicotine levels was drawn pre-dose and 10, 20, 30, 40, 50 min, 1, 1.5, 2, 2.5, 3, 3.5, 4, 5, 6, 7, 8, 9, 10, 11, 13, 16 and 48 h post-dose.

Criteria for safety evaluation were as follows: (1) C_max _and t_max _after each dose, as well as AUC_10 min-12 h _for nicotine PK; (2) BP, HR, ECG, and physical examination for clinical status; (3) hematology, biochemistry (including hepatic parameters, serology and urinalysis), carboxyhemoglobin, carcinoembryonic antigen, and urinary cotinine and catecholamine levels for laboratory testing; and (4) gastric emptying and antral motor activity for gastric motility. Nicotine PK parameters were analyzed by summary statistics of all nicotine concentrations, AUC, t_max_, C_max_, and comparison of PK values by one-way ANOVA. BP and HR were analyzed by an ANOVA GLM model for systolic and diastolic values, as well as for the difference (systolic – diastolic); factors for ANOVA were subject, period and treatment. All other parameters were analyzed within each treatment group by Wilcoxon paired test (pre- vs. post-dosing). Post-entry differences of each treatment group were compared by one-way ANOVA or Kruskal-Wallis test, as appropriate. For all parameters, McNemar test was used to detect volunteers who changed their status from entry to post-dosing in terms of normal range limits.

### Efficacy trials with 1 mg lozenge

Two large phase 3 studies were performed to assess the efficacy of 1 mg Nicotinell lozenge in smoking cessation, one in France (37 centers; AFSSAPS trial registration number: 980324) and one in the USA (3 centers; registration number 905.1295). The 2 trials had a double-blind, placebo-controlled, randomized, parallel groups design. The patients, respectively for the French and the American trials, were distributed as follows: (1) randomized and received at least one treatment: 436 and 460 patients, (2) received active lozenge: 214 and 230 patients, (3) received placebo lozenges: 222 and 230 subjects. Eligible subjects were healthy smokers of both sexes and aged ≥ 18 years, motivated to quit. To have a global view on the lozenge efficacy over a wide range of dependence levels, patients had either a medium to high dependence of smoking in the French study (Fagerström Test for Nicotine Dependence (FTND) [24] score of 4–7 and smoking 10–30 cigarettes/day), or a high to very high dependence in the US study (FTND score ≥ 6 and smoking 20–40 cigarettes/day). Subjects were advised to suck between 8 and 25 lozenges each day (dosage self-adjusted by subjects) for six weeks, followed by complete weaning over the next six weeks with a suggested decrease of one lozenge per day relative to the dose at week 6. Subjects attended the study site at screening (baseline), biweekly or weekly up to week 6, and then at week 12, and 26. After week 6, only abstinent subjects were continuously followed-up. At visits, smoking status (exhaled-breath CO), BP, HR, weight, and AEs were recorded. From weeks 0–6 (France) or 0–12 (USA), subjects recorded daily in a diary the number of lozenges used and of cigarettes smoked (if any), plus any withdrawal symptoms experienced. During the study no formal behavioral counseling was offered to patients. The primary efficacy outcome measure was the proportion of patients continuously and completely abstinent (no cigarettes and CO <10 ppm) during the four weeks preceding week 6 and the number of subjects to include was calculated according to this objective. Secondary efficacy outcomes were the point prevalence abstinence rates at the visits on week 12 and on week 26, the evolution of withdrawal symptom scores, and partial abstinence rates (partial abstinence defined as smoking of ≤ 1 cigarette per day and ≤ 7 cigarettes per week, and CO <10 ppm) on week 6, 12 and 26. Safety of treatment (AEs, BP, HR, weight) and levels of urine nicotine metabolites (French study only) were further outcome measures. Efficacy was analyzed using Cochran-Mantel-Haenszel statistic, stratified by study center. Comparison between treatments was made using the Cochran-Mantel-Haenszel chi-square test, after adjusting for pooled centers. The experimental details and results of the French trial have previously been described [17,18].

## Results

### Single dose pilot study: PK comparison of 1 and 2 mg lozenges with 2 mg gum

Key PK results are summarized in Table 2. The sucking pattern in this trial did not result in full dissolution of the lozenges and only approximately 67% of their nicotine content was released. Thus, a correction was applied to calculate C_max _and AUC_0–∞ _for a fully dissolved lozenge. This was deemed appropriate because lozenges are designed to be fully sucked out by users. The adjusted values for C_max _and AUC_0–∞ _of 1 mg lozenge and 2 mg gum were similar and thus indicated comparable nicotine delivery from these two formulations. In addition, comparison of PK parameters of 1 and 2 mg lozenges demonstrated dose-concentration proportionality between these doses, which was verified in an ensuing multiple-dose study (see below).

**Table 2:** PK parameters of nicotine in plasma after intake of a single dose of lozenge or gum (mean ± SD; n = 9)

**Formulation\PK parameter**	**Nicotinell^® ^** **lozenge**		**Nicorette^® ^** **gum**
		
	**1 mg**	**2 mg**	**2 mg**
**Amount of nicotine released after 30 min (mg) ** [% of minimal content]	0.67 ± 0.14 [67%]	1.39 ± 0.28 [67%]	0.81 ± 0.25 [42%]
**observed C_max _(ng/ml) ** [adjusted]^†‡^	2.3 ± 0.8 [3.5 ± 1.1]^†^	4.8 ± 1.4 [7.0 ± 1.7]	2.9 ± 1.2
**t_max _(h) ** [median (range)]	1.1 ± 0.7 [0.8 (0.8–3.0)]	0.8 ± 0.2 [0.8 (0.6–1.1)]	0.8 ± 0.1 [0.8 (0.5–1.0)]
**t_1/2 _(h)**	2.7 ± 0.7	2.8 ± 0.7	2.5 ± 1.0
**AUC_0-t _(h·ng/ml)**	8.3 ± 2.5	15.8 ± 4.1	10.6 ± 4.4
**observed AUC_0–∞_(h·ng/ml) ** [adjusted]^†‡^	10.7 ± 3.1 [16.5 ± 4.6]	20.0 ± 5.9 [30.1 ± 9.7]	13.8 ± 5.6

†: Since in this trial nicotine lozenges were not completely dissolved after 30 min of sucking, an adjustment was made for nicotine delivery by a full 1 mg dose.

‡: Adjustment for nicotine delivery by a full 2 mg dose.

During metabolization, nicotine is converted into several metabolites including cotinine, which are finally excreted in urine. Further support for comparable PK properties of 1 mg lozenge and 2 mg gum came from mean plasma cotinine kinetics, including C_max _(19.9 → adjusted 29.7 ng/ml with lozenge vs. 30.2 ng/ml with gum), AUC_0-t _(102 → 152 h.ng/ml vs. 153 h.ng/ml), t_max _(5.2 vs. 5.3 h), and t_1/2 _(9.4 vs. 11.4 h). Similarly, urinary cotinine values were comparable between 1 mg lozenge and 2 mg gum, and dose-concentration relationship was apparent for urinary cotinine excretion after dosing with 1 mg vs. 2 mg lozenges (data not shown). Among the 18 AEs observed, only 2 were considered to be related to study drug (throat irritation with 2 mg lozenge and gum), but none was serious. No other clinically relevant abnormalities were noted with any of the products.

### Bioequivalence of 1 mg lozenge and 2 mg gum at steady state

As the pilot study suggested bioequivalence of 1 mg lozenges and 2 mg gum, this trial was designed to confirm this bioequivalence between these two formulations, focusing on PK characteristics after multiple doses and at steady state. Median duration for complete dissolution of lozenges over the entire study period was 29.25 min (range 27–31 min). The mean amount of nicotine extracted from the gum increased from 1.0 mg at dose 1 to 1.13 mg at dose 12 (+13%). Therefore, an adjustment was applied to the amount of nicotine actually delivered by gum at different time-points in order to ensure consistent statistical analysis of PK parameters. Both NRT products led to an increase in saliva pH after dosing, the lozenge having a significantly higher buffering power than the gum (data not shown), which is known to favor a non-ionized state of nicotine and its absorption [25,26]. As expected, median t_max _(0.76 vs. 0.75 h), mean C_max _(4.2 ± 1.8 vs. 5.0 ± 1.6 ng/ml) and AUC_0–1 _(2.9 ± 1.4 vs. 3.5 ± 1.3 h.ng/ml) were similar for lozenge and gum after the first dose (0–1 h).

**Figure 1:**
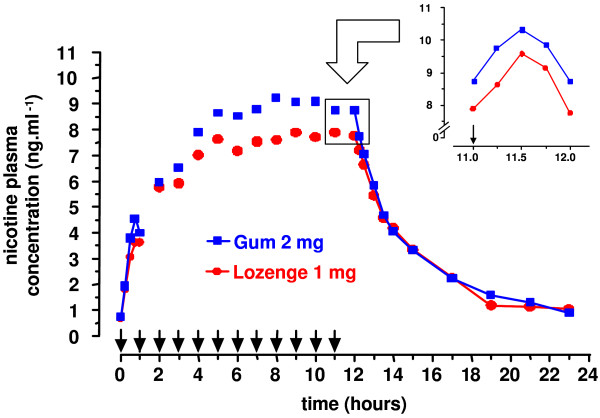
**Nicotine plasma concentration after repeated intake (24 subjects)**. Nicotine plasma concentrations were determined in this multiple-dose bioequivalence trial with 24 volunteers from blood samples drawn before administration of each single dose (unconnected data points). Consecutive blood samples (connected data points) were drawn after administration of dose 1 (t = 0) and dose 12 (t = 11 h). The insert shows a blood nicotine peak after dosing at steady state obtained with blood draws after dose 12 (11–12 h).

Plotting of plasma nicotine concentrations over time after repeated administration of Nicotinell 1 mg lozenges and 2 mg polacrilex gum revealed that the plateau of concentration was reached after intake of six doses (Figure 1).

The blood draws after the last dose allowed to determine PK characteristics at steady state. The observed kinetics suggested that intake of lozenges or gum under these conditions lead to "peak-trough" kinetics of nicotine similar to those observed during day-time in dependent smokers having cigarettes, but much reduced in intensity. Indeed, the highest concentration (approximately 11 ng/ml) obtained with the tested formulations corresponds to the lower end of the range of blood nicotine levels (10–50 ng/ml) observed in smokers in the afternoon (i.e. at steady state) [14].

The determined values for C_max_, t_max _and AUC_11–12 _showed that nicotine delivery characteristics of 1 mg Nicotinell lozenge and 2 mg polacrilex gum at steady state were comparable (Table 3). When bioequivalence was statistically assessed, the 90% CIs for C_max _and AUC_11–12 _remained within the standard BAR [0.80–1.25], indicating bioequivalence of the 1 mg lozenge and the 2 mg gum under the studied conditions. When unadjusted values for gum were considered, the 90% CIs for C_max _and AUC_11–12 _of lozenge (0.76–0.93 and 0.75–0.91, respectively) still fell within the enlarged BAR [0.7–1.43], which may be considered acceptable for oral nicotine, due to its broad therapeutic range and self-titration behavior of users [27-29].

**Table 3:** PK parameters of nicotine in plasma at steady state after 12 hourly doses^† ^of 1 mg lozenge or 2 mg gum (mean ± SD; n = 24)

**Formulation\PK parameter**	**Nicotinell^® ^lozenge**	**Nicorette^® ^gum**	**90% CI **(lozenge/gum)^#^
		
	**1 mg**	**2 mg^‡^**	
**C_max _(ng/ml)**	10.6 ± 2.9	11.4 ± 3.8	0.86–1.04
**C_min _(ng/ml)**	8.1 ± 2.4	9.3 ± 3.4	ND
**t_max _(h) **[range]	0.54 ± 0.21 [0.00–0.77]	0.47 ± 0.19 [0.20–0.95]	ND
**t_1/2 _(h)**	3.6 ± 1.2	3.2 ± 0.9	ND
**AUC_11–12 _(h·ng/ml)**	9.2 ± 2.6	10.2 ± 3.4	0.84–1.01

Initial nicotine concentrations in plasma were 1.1 ng/ml (range: 0.0–6.5) with lozenge and 0.9 ng/ml (0.0–5.1) with gum, reflecting volunteers' heavy smoking habits (≥ 20 cigarettes per day). Correction for initial nicotine concentrations did not modify the results and thus was not retained. ND: not determined.

†: PK parameters were determined from blood samples drawn after dose 12 (t = 11 h) until t = 23 h.

‡: a dose adjustment of the raw data was done for a last standard dose of 1 mg nicotine delivery from the gum.

#: Standard 90% CI for the expected mean ratio was derived from ANOVA for continuous parameters and compared to standard BAR [0.80–1.25].

With respect to safety, 12 volunteers had a total of 40 AEs, which were all mild to moderate, with complete recovery. Six AEs (2 for lozenge and 4 for gum) were considered as drug-related: headache, salivation, hiccups, flatulence and throat irritation. No other clinically relevant abnormalities were observed, neither for cardiovascular parameters, nor for laboratory testing.

### Dose proportionality between 1 and 2 mg lozenges and comparison with 4 mg gum at steady state

**Figure 2:**
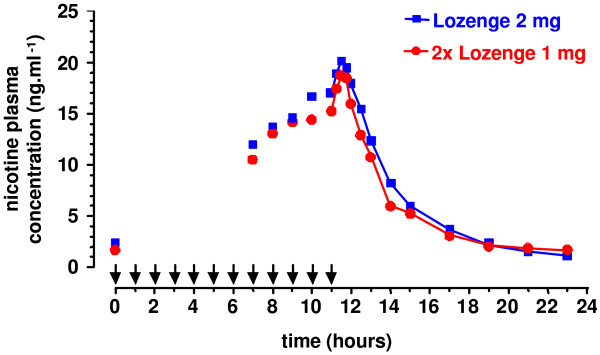
**Dose proportionality of nicotine lozenges with respect to nicotine plasma concentrations (31 subjects)**. Nicotine plasma concentrations were determined from blood samples of 31 volunteers drawn before hourly administration 1 or 2 mg nicotine lozenge (unconnected data points). Consecutive blood samples (connected data points) were drawn after administration of dose 12 (t = 11 h), and the derived data points are connected. Plasma concentrations obtained with 1 mg lozenges were doubled for ease of comparison.

This study was performed to confirm the dose-concentration proportionality between 1 and 2 mg Nicotinell lozenges, and to compare the nicotine PK profile of lozenges with that of 4 mg nicotine gum. Overall trial design was comparable to the previous multiple-dose study, with intake of 12 doses of nicotine by 31 volunteers (1 dose per hour, 30 min of sucking or standardized chewing) and assessment of plasma nicotine PK parameters at steady state (successive blood draws after dose 12). Thirteen out of 31 (42%) of all trial participants had significant baseline plasma nicotine levels on two or three periods, but no differences were observed between the three treatment groups. Thus, these baseline levels were not taken into account, as they did not influence the comparison between groups. The plateau concentration for plasma nicotine was reached before the 12th dose. With respect to dose-concentration proportionality between the 1 and 2 mg lozenge, an adjustment for the same dose (2 mg) was applied, and C_max _and AUC_11–12 _were determined (Table 4). As expected, the 90% CIs of the ratio of C_max _and AUC_11–12 _fell within the standard BAR [0.80–1.25], thus demonstrating a linear dose-concentration relationship between the two strengths of nicotine lozenge (Figure 2). In addition, t_max _did not to differ significantly (Friedman ANOVA).

**Table 4:** Steady state^† ^plasma nicotine PK parameters after intake of 1 or 2 mg lozenges to determine dose-concentration proportionality (mean ± SD; n = 31)

**Formulation\PK parameter**	**Nicotinell^® ^lozenge 1 mg**		**Nicotinell^® ^lozenge**	**90% CI **(2 mg/1 mg)^#^
		
	**observed**	**adj. to 2 mg^‡^**	**2 mg**	
**C_max _(ng/ml)**	11.0 ± 4.9	22.0 ± 9.8	22.5 ± 7.0	0.97–1.19 (F = 1.07)
**t_max _(h) **Median [range]	0.5 [0.25–1.00]	-	0.5 [0.25–1.02]	NS (Friedman ANOVA)
**AUC_11–12 _(h·ng/ml)**	9.7 ± 3.9	19.3 ± 7.8	20.2 ± 6.8	0.98–1.20 (F = 1.08)

NS: not significant.

†: PK parameters were determined from blood samples drawn after dose 12 (t = 11 h) until t = 23 h.

‡: Experimental values (plasma nicotine concentrations) observed with 1 mg lozenge were adjusted to a hypothetical 2 mg dose before calculation of PK parameters.

#: Standard 90% CI for the expected mean ratio was derived from ANOVA for continuous parameters and compared to standard BAR [0.80–1.25]. F: relative bioavailability ratio.

**Figure 3:**
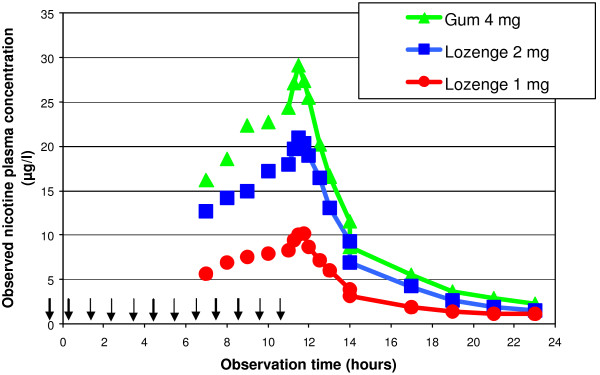
**Nicotine plasma concentration after repeated intake of nicotine lozenges or gum (31 subjects)**. Nicotine plasma concentrations were determined from blood samples of 31 volunteers drawn before hourly administration of 1 or 2 mg nicotine lozenge or 4 mg nicotine gum (unconnected data points). Consecutive blood samples (connected data points) were drawn after administration of dose 12 (t = 11 h).

As bioequivalence between 1 mg lozenges and 2 mg gum had been shown (see above) and as 1 and 2 mg lozenges showed dose proportionality, one of the aims of this study was to compare 2 mg lozenge with 4 mg gum. With 2 mg lozenges, C_max _and AUC_11–12 _were approximately 26% lower than with 4 mg gum. No bioequivalence was observed (Table 5, Figure 3).

**Table 5:** PK parameters of nicotine in plasma at steady state after 12 hourly doses^† ^of 2 mg lozenge or 4 mg gum (mean ± SD; n = 31)

**Formulation\PK parameter**	**Nicotinell^® ^lozenge**	**Nicorette^® ^gum**	**90% CI **(lozenge/gum)^‡^
		
	**2 mg**	**4 mg**	
**C_max _(ng/ml)**	22.5 ± 7.0	30.5 ± 12.8	0.69–0.89 (F = 0.78)
**t_max _(h) **Median [range]	0.5 [0.25–1.02]	0.5 [0.25–1.00]	NS (Friedman ANOVA)
**AUC_11–12 _(h·ng/ml)**	20.2 ± 6.8	27.5 ± 11.4	0.68–0.88 (F = 0.77)

NS: not significant.

†: PK parameters were determined from blood samples drawn after dose 12 (t = 11 h) until t = 23 h.

‡: Standard 90% CI for the expected mean ratio (test/reference = lozenge/gum) was derived from ANOVA for continuous parameters and compared to standard BAR [0.80–1.25] and enlarged BAR [0.7–1.43]. F: relative bioavailability ratio.

Analysis of safety and tolerability was considered of particular value in this trial, as participants were treated with a wide range of nicotine doses under consistent conditions. Overall, no significant variations were reported in physical examination, vital signs, cardiovascular parameters or clinical laboratory tests. There were no serious or unexpected AEs. One subject was withdrawn due to vomiting when taking the 2 mg lozenges. The relationship was considered probable. Sixteen subjects reported a total of 29 AEs during the entire trial, which were all mild to moderate. The most frequent AEs assessed as at least possibly related to treatments were: hiccup (reported by 10 subjects, nine of which under the gum), throat irritation (two times with 2 mg lozenges and three times with gum), and nausea (ditto). Globally, more AEs were reported with the 4 mg gum than with the 1 or 2 mg lozenges, suggesting overall good tolerability of the latter formulation.

### Safety in case of misuse of 1 mg lozenges

All clinical assessments performed in the PK trials, as well as reported AEs, suggested a favorable safety profile for Nicotinell^® ^lozenges. However, in these trials one lozenge at a time was administered and subjects were sucking them slowly without concomitantly smoking. Yet, nicotine from oral formulations is not only taken up via the oral mucosa, but is also partially absorbed from the gastrointestinal tract [26]. To assess the safety in case of swallowing large quantities of 1 mg lozenges, an escalating-dose safety open study was performed: three groups (A, B, C), each including eight male or female subjects who were at least moderate smokers (≥ 10 cigarettes/day) received either 3, 6, or 12 Nicotinell 1 mg lozenges, to be swallowed at once with water. Smoking abstinence prior to dosing was not asked for and all of the volunteers (except one) had measurable levels of nicotine in plasma before lozenge intake. Blood was drawn until 48 h post-dose and plasma nicotine PK parameters were determined (Table 6). The highest mean concentration observed (20.5 ± 8.8 ng/ml) under these experimental conditions of misuse still fell largely within the range of plasma nicotine concentrations observed in active cigarette smokers (10–50 ng/ml) [14]. Of note, these observed concentrations were not corrected for the considerable baseline nicotine levels of the subjects.

**Table 6:** PK parameters of nicotine in plasma after swallowing without sucking of increasing numbers of 1 mg lozenge (mean ± SD; n = 8 per group)

**Group\PK parameter**	**A**	**B**	**C**
	
	**3 lozenges**	**6 lozenges**	**12 lozenges**
**C_max _(ng/ml)**	8.2 ± 7.6	16.9 ± 11.8	20.5 ± 8.8
**t_max _(h)**	2.7 ± 2.8	2.8 ± 2.0	2.2 ± 1.4
**AUC_10 min-12 h _(h.ng/ml)**	37.0 ± 27.2	84.9 ± 48.9	103.1 ± 58.6

PK parameters were not corrected for baseline plasma nicotine levels.

The safety of lozenges when misused was also monitored through the assessment of the clinical status (including cardiovascular parameters), laboratory test results and occurrence of AEs. The different extents (according to dose) of nicotine absorption were not reflected in BP, HR, and ECG values, nor were any clinically significant changes in cardiac or laboratory parameters observed. No serious AEs were reported. Only one minor AE occurred in groups A and B (headache during 1 h). In group C, six volunteers reported transient stomach heaviness lasting for up to 1 h after ingestion of the 12 lozenges. Gastric antral motor activity determined at 1 and 11 h post-dosing was identical to normal physiological values (3 ± 0.5 cycles/min). Gastric half clearance times at 1 and 11 h post-dosing were within the standard range (55 ± 15 min) for subjects receiving 3 or 6 lozenges, but not for those receiving 12 lozenges; the latter complained about stomach heaviness at 1 h post-dosing and displayed shortened gastric half clearance times (26 and 34 min). However, these AEs resolved spontaneously, and clearance half-times at 11 h post-dosing were again well within the standard range (49 and 62 min).

### Efficacy of 1 mg lozenge for smoking cessation in phase 3 trials

The two randomized placebo-controlled trials to assess the clinical efficacy of 1 mg Nicotinell lozenge, involved approximately 900 subjects. For both trials, the demographic characteristics of enrolled subjects, including parameters related to past smoking history (years of smoking, number of cigarettes per day, exhaled CO, and urinary nicotine metabolite levels) were matched between groups (data not shown). The only important difference between the two studies was the level of patients' cigarette dependence at inclusion (see Methods).

**Figure 4:**
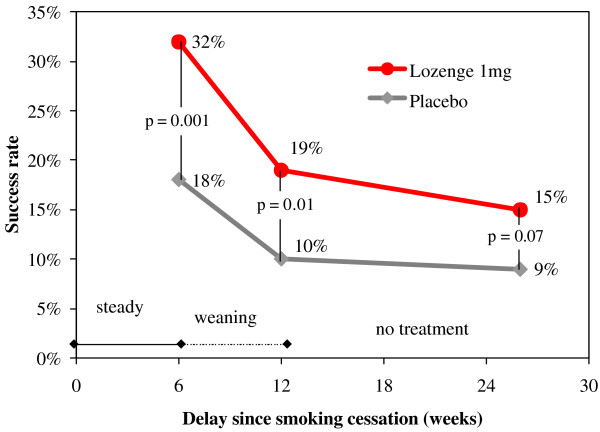
**Smoking cessation success rates among users of 1 mg nicotine lozenges (moderately dependent patients) (adopted from [17])**. The rate of fully (no cigarettes) or partially (≤ 1 cigarette/day and ≤ 7 cigarettes/week) abstinent subjects using 1 mg nicotine lozenges for abrupt quitting over the first 6 months of the French efficacy trial.

**Figure 5:**
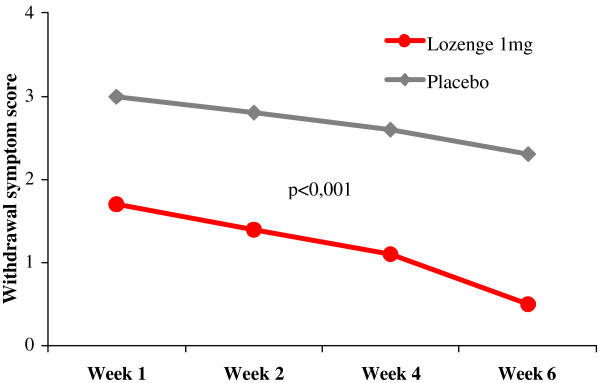
Withdrawal symptom score (change vs. baseline) among users of 1 mg nicotine lozenges (adopted from [18]).

The results for the primary efficacy criterion of the French trial on week 6 indicated a statistically significant increase in the number of subjects continuously abstinent from smoking with active lozenges, with an odds ratio (OR) for abstinence of 1.72 [95% CI: 1.05–2.80] compared with placebo (Table 7). This result was in line with previously reported odds for quitting with NRT products in general [9] and a polacrilex lozenge formulation 2 mg in particular [16]. The full numbers about complete abstinence from week 2 are detailed in table 8. When the less selective, secondary efficacy criterion was applied (complete or partial smoking abstinence on week 6), the OR for abstinence with the active lozenge was 2.18 [1.40–3.41], and statistically significant differences (p < 0.05) between active and placebo lozenges in the percentage of abstinent smokers were found not only at week 6, but also for the follow-up visit at week 12 (Table 7 and Figure 4). On week 26, this difference was only significant for the subgroup of subjects previously smoking >20 cigarettes per day (data not shown). In addition, the evolution (compared to baseline) of the score for cessation-related withdrawal symptoms was determined, and increases proved to be less intense for subjects randomized to the active group (p < 0.001) than for those in the placebo group (Figure 5). Finally, there was no increased frequency of any side effects in the active group and vital signs control did not reveal any relevant differences between the two treatment groups.

**Table 7:** Primary and secondary efficacy criteria: complete or partial abstinence during the 28 days preceding the 6-week visit (adopted from [17])

**Group\Statistical results**	**Nicotinell^® ^** **lozenge 1 mg**	**Placebo**
	
	**(n = 211)**	**(n = 222)**
**Complete abstinence (%)**	48 (23%)	32 (14%)
**OR **[95% CI]	**1.72 [1.05–2.80]**	
**Statistical significance**	**p = 0.03**	
**Complete or partial abstinence (%)**	68 (32%)	39 (18%)
**OR **[95% CI]	**2.18 **[1.40–3.41]	
**Statistical significance**	**p = 0.001**	

Complete abstinence = subjects declaring not to smoke + exhaled-breath CO <10 ppm. Partial abstinence = subjects smoking ≤ 1 cigarette/day and ≤ 7 cigarettes/week + exhaled-breath CO <10 ppm.

**Table 8:** Complete abstinence from week 2 at each visit

**Time since treatment started**		**6 weeks**		**12 weeks**		**26 weeks**	
**French study (FTDN 4 to7)**		Placebo n = 222, Nicotinell Lozenge 1 mg n = 211					

**Completely abstinent**	active	48	**22.7%**	28	**13.3%**	25	**11.8%**
**N (%)**	placebo	32	**14.4%**	20	**9.0%**	18	**8.1%**
**OR **[95% CI]		**1.72 **[1.05–2.80]		**1.47 **[0.82–2.65]		**1.48 **[0.78–2.79]	
**Statistical significance**		**p = 0.03**		**p = 0.199**		**p = 0.227**	

**USA study (FTND ≥ 6)**		Placebo n = 230, Nicotinell Lozenge 1 mg n = 230					

**Completely abstinent**	active	19	**8.3%**	11	**4.8%**	8	**3.5%**
**N (%)**	placebo	7	**3.0%**	3	**1.3%**	2	**0.9%**
**OR **[95% CI]		**2.87 **[1.18–6.97]		**3.80 **[1.05–13.80]		**4.11 **[0.83–19.55]	
**Statistical significance**		**p = 0.02**		**p = 0.03**		**p = 0.06**	

Completely abstinent = subjects declaring not to smoke + exhaled-breath CO <10 ppm

**Figure 6:**
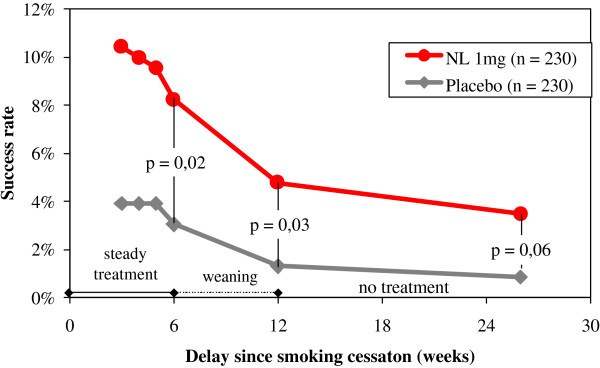
**Smoking cessation success rates among users of 1 mg nicotine lozenges (highly dependent patients)**. The rate of completely abstinent subjects using 1 mg nicotine lozenges for abrupt quitting over the first 6 months of the U.S. efficacy trial.

In the US trial, analysis for the primary efficacy criterion at week 6 also demonstrated a statistically significant superiority for active vs. placebo lozenges in the number of quitters who remained continuously abstinent on week 6 (OR = 2.87 [95% CI: 1.18–6.97]) (Table 8). The proportion of subjects continuously abstinent from week 2 through to week 12 was also significantly higher with active lozenge than with placebo lozenge (Table 8, Figure 6). However, the abstinence rates were lower than in the French trial, which might be due to patients' higher dependence levels at inclusion. Withdrawal symptoms, assessed as total craving and total mood scores, were reduced in the active group in comparison with the placebo group during the steady treatment period (up to week 6), with statistically significant differences in mean total craving scores at week 1 (p = 0.03), and weeks 2 and 3 (p < 0.01).

Overall, Nicotinell 1 mg lozenges significantly increased the ratios of short-term abstinent from smoking patients, with no increase in side effects compared with placebo. Of interest, the mean consumption in the US trial, which included more highly dependent smokers than the French trial, consisted of 12.9 nicotine lozenges/day during the steady treatment phase (week 1–6), and decreased from 8.8 lozenges/day (week 7) to 3.3 lozenges/day (week 12) during the weaning phase. Corresponding amounts of lozenges/day in the French trial were 8.7 (week 1–6), 6.2 (week 7), and 1.9 (week 12). These numbers provided evidence for the self-titration behavior of smokers when using Nicotinell^® ^1 mg lozenge, with subjects adjusting their intake to their individual needs.

## Discussion

This review presents PK data obtained during the clinical development process of 1 and 2 mg Nicotinell lozenges, which show that these formulations deliver significant amounts of nicotine, despite lower amounts of nicotine-base equivalents compared to the polacrilex 2 mg chewing gum. In addition, we present clinical data, which demonstrate that these lozenges are effective and safe for use by smokers wanting to quit smoking.

The high addictiveness of tobacco smoking makes it difficult to quit without some form of additional help [30,31]. Pharmaceutical NRT, i.e. the administration of purified nicotine under controlled regimens to smokers in order to help them to stop smoking cigarettes, aims at alleviating craving for nicotine and thereby preventing rapid relapse. Accordingly, over the last 20 years, a body of experimental evidence has demonstrated that the chances for motivated smokers to successfully quit were roughly doubled when NRT products were used [9]. Nicotine for NRT is available in a number of different pharmacological formulations, and lozenges are among the latest additions to the arsenal of NRT products [16,22]. Oral nicotine formulations present both advantages and inconveniences directly related to this route of administration: their major drawback, as compared with transdermal patches, is that repeated intake of the drug is necessary to achieve sustained blood nicotine levels. On the other hand, the versatility of acute dosage forms allows abstinent smokers to address temporary craving episodes, and gives them the possibility of a behavioral response somehow similar to the previous cigarette-linked gestures.

Oral nicotine formulations are the treatment of choice when abstinent smokers want to keep an active control in situations that may trigger smoking relapse, e.g. before stressful meetings or social events [8,32]. While worldwide the gum formulations are the most common oral NRT products, a number of disadvantages might render people reluctant to use them: incompatibility with dental works or temporomandibular joint pain, cultural or social acceptance and occurrence of AEs (e.g. hiccup or throat irritation) [33]. In this respect, lozenges present a valuable alternative as their use is more discrete and aesthetic.

Nicotine is best absorbed when it is in its non-ionized form, which rapidly crosses biomembranes. Accordingly, in the development of 1 and 2 mg Nicotinell lozenges, particular attention has been paid to optimization of the alkalizing resin to obtain optimal nicotine release and absorption. The new formulations for lozenges were compared to 2 and 4 mg nicotine polacrilex gum (Nicorette^®^), which are pharmacologically well-characterized and were the first oral NRT product on the market [15,26,34,35].

Comparison of PK parameters after a single dose of lozenge or gum showed that the amount of nicotine absorbed through 1 mg Nicotinell lozenges is similar to that obtained via 2 mg polacrilex gum, and that both formulations have comparable kinetics. In addition, dose-concentration proportionality between 1 and 2 mg Nicotinell^® ^lozenges was evident from PK results, with a net doubling of C_max _and AUC achieved with the 2 mg formulation compared to the 1 mg formulation. The discrepancy in gross nicotine delivery between Nicotinell 1 mg lozenges and polacrilex 2 mg gum in the single-dose study is only apparent and was not found in the latter studies, when the sucking pattern was adapted: lozenges are designed to be completely sucked out, whereas gum are known to retain a significant amount of nicotine within the resin (28% and 47% of total nicotine content, respectively for 4 mg and 2 mg gum, i.e. approximately 1 mg nicotine for both strenght) [25]. Besides, variations among individuals, probably due to differences in mastication intensity, chewing techniques or oral pH could explain some of the differences [26].

Comparable absorption of nicotine from 1 mg lozenges and 2 mg gum was confirmed in the first multiple dose trial, performed to compare the PK profile of both formulations under conditions close to real use of NRT products. At steady state, all assessed PK parameters were similar and formal bioequivalence using the standard BAR [0.8–1.25] was demonstrated for C_max _and AUC. In the second multiple dose trial the dose-concentration proportionality between Nicotinell^® ^1 and 2 mg lozenges was confirmed, with 2 mg lozenges inducing plasma concentrations intermediate between those obtained with 1 mg lozenges and 4 mg polacrilex gum.

Accordingly, the systemic exposure to nicotine with Nicotinell lozenges and with Nicorette gum, under conditions reflecting the intended use, could be ranked as follows: 4 mg polacrilex gum > 2 mg Nicotinell lozenge > 1 mg Nicotinell lozenge = 2 mg polacrilex gum.

The criteria applied to evaluate bioequivalence between different NRT products can give rise to discussion. Indeed, even when the standard BAR [0.8–1.25] does not argue in favor of bioequivalence, the enlarged BAR [0.7–1.43] is relevant to assess bioequivalence between two oral NRT formulations. This reasoning is based on the particular behavior encountered in smokers: smokers regulate their intake of nicotine by the number of cigarettes they smoke and their inhaling mode. They achieve the desired effects and minimize withdrawal symptoms by adjusting an individual "optimal" blood level of nicotine [27]. Similarly, the intake of NRT oral or nasal products is subject to self-titration: clinical studies showed that the use of nicotine gum or nasal spray followed a principle of self-titration similar to that observed with cigarettes, with an ad-libitum schedule resulting in more constant blood levels than a fixed schedule [28,29]. It can reasonably be assumed that nicotine self-titration behavior is found with all cessation products administered in multiple doses per day and favors individual adjustment of the dose to achieve the desired effect. In this respect, bioequivalence using the enlarged BAR [0.7–1.43] was achieved in the multiple-dose study comparing 1 mg lozenge with 2 mg gum, even when no adjustment for increasing nicotine release from the gum with time was applied (90% CI [lozenge/gum] for C_max_: 0.76–0.93; for AUC_11–12_: 0.75–0.91).

The safety of 1 and 2 mg Nicotinell lozenges can be assessed based on the observations of the 70 healthy adult male smokers enrolled in the PK trials. In this study population exposed to single or multiple doses of lozenges, no serious or unexpected AEs and no changes in physical examination, vital signs, cardiovascular parameters and laboratory values were observed. Reported AEs were mild or (at worst) moderate in intensity, transient and completely reversible. This good tolerability of lozenges is in line with safety results reported in previous clinical trials with nicotine-polacrilex lozenges [16,22,36]. Even during the complementary safety trial, with subjects swallowing up to 12 lozenges at once, no serious or lasting AEs were reported and the overall tolerability was rated as excellent. Finally, the data from the two large phase 3 efficacy trials further confirmed the safety profile of the nicotine lozenges, as there were no relevant differences in AE frequency or nature between placebo and active groups. In addition, the vast majority of the reported AEs could also be attributed to the effects of abrupt smoking cessation.

The efficacy trials were designed to provide sufficient power for the statistical analysis of the primary efficacy criterion: controlled continuous smoking abstinence during the 28 days preceding the visit scheduled at week 6. This primary efficacy criterion was severe, as it obliged subjects to stop smoking completely before the end of the second week of treatment, compared with 3-week to 12-month reported for some nicotine gum trials [9]. However, the short treatment period seems to be more in line with "real world" use by consumers [37]. In addition, the weaning phase of 6 weeks (from week 7 to 12, after which no more lozenges were supplied) was also relatively short compared with some published nicotine gum trials, and subjects starting to smoke again were excluded from follow-up, regardless of their ensuing performance. These particularities must be remembered when interpreting the efficacy results. Further, in both trials, no specific behavioral counseling or support was provided to the studied populations. Notwithstanding these facts, the statistically significant differences in short-term abstinence rates as well as the odds for successful quitting with lozenge vs. placebo did underscore the efficacy of 1 mg nicotine lozenge as a smoking cessation aid.

Comparative analysis of PK results of the 1 and 2 mg lozenge showed that at steady state C_max _was approximately 11 and 23 ng/ml, respectively. Notably, these levels are on the lower end of the range of blood nicotine levels (10–50 ng/ml) observed in smokers at steady state. As nicotine underdosing during smoking cessation is generally considered to increase the risk for failure in nicotine dependent patients [13,37], the 2 mg Nicotinell lozenge may provide increased efficacy for heavily to very heavily dependent smokers, whereas the 1 mg dose may fulfill the needs of light to moderate dependent smokers. Considering a possible abuse potential of the lozenges, which is inherent to all NRT products administered in multiple doses per day, it should be noted that in the French efficacy trial urinary cotinine levels decreased not only in the population of abstinent subjects receiving placebo, but also in the population receiving 1 mg lozenges, thus clearly indicating that at least short-medium term quitters do not overdose the product.

## Conclusion

The data presented in this review demonstrate high nicotine bioavailability, excellent safety profile and proven short-term efficacy of Nicotinell lozenges. At nominal equivalent doses 1 and 2 mg Nicotinell lozenges were shown to deliver larger amounts of bioavailable nicotine compared to the nicotine-polacrilex gum. Thus, Nicotinell 1 mg lozenge was bioequivalent to 2 mg polacrilex gum following single as well as multiple doses, whereas the 2 mg lozenge provided nicotine levels between those of the 2 and the 4 mg polacrilex gum. Accordingly, the systemic exposure to nicotine could be ranked: 4 mg polacrilex gum > 2 mg Nicotinell lozenge > 1 mg Nicotinell lozenge = 2 mg polacrilex gum

All AEs observed during the clinical development were mild or moderate in severity, transient and completely reversible. With respect to efficacy in smoking cessation, significantly higher short-term abstinence rates (continuous abstinence from week 2, assessed on week 6) were achieved with lozenge compared to placebo. In conclusion, Nicotinell lozenges offer a valuable addition to the therapeutic armamentarium available for smoking cessation.

## Competing interests

J.-L. Kienzler and A. Callens are employees of Novartis Consumer Health S.A. which produce and distribute nicotine replacement products. All research trials were funded by Novartis Consumer Health.

## Authors' contributions

BD was the principal investigator of the French phase 3 efficacy trial. MN participated to the Novartis Consumer Heath smoking cessation activities in the US. JLK and AC supervised and coordinated the clinical development of the Nicotinell^® ^lozenges. All authors read and approved the final manuscript.

## Pre-publication history

The pre-publication history for this paper can be accessed here:


